# Community-Acquired Meningitis Complicated With Pyogenic Ventriculitis and Hydrocephalus in a Patient With Haematological Malignancy: A Case Report and Literature Review

**DOI:** 10.7759/cureus.60800

**Published:** 2024-05-21

**Authors:** Anosh Aslam Khan, Muhammad Saad Anwar, Phani Bhavana Cherukuri, Amer Abu-Shanab, Peter N Fish

**Affiliations:** 1 Department of Internal Medicine, Monmouth Medical Center, Long Branch, USA

**Keywords:** meningitis treatment, immunocompromised, hydrocephalus, ventriculitis, pneumococcal meningitis

## Abstract

Meningitis, an infection of the meninges of the central nervous system (CNS), can advance quickly and carries a mortality rate reaching 30% among affected patients. It may become complicated by conditions such as hydrocephalus, ventriculitis, and cerebral abscess. Here, we describe a case of meningitis that was complicated by pyogenic ventriculitis and hydrocephalus in a patient with diffuse large B-cell lymphoma (DLBCL) who underwent chemotherapy and radiotherapy. The patient presented with acute change in mental status and high-grade fever, with few episodes of non-bloody vomiting. Blood culture and cerebrospinal fluid (CSF) culture grew *Streptococcus pneumoniae,* which was sensitive to ceftriaxone. CT scan of the head showed ventriculomegaly, pansinusitis, and a large left mastoid effusion. MRI of the brain showed layering in ventricles, hydrocephalus, and dural enhancement consistent with pachymeningitis. She was treated with ceftriaxone for 21 days with a meaningful outcome. She was discharged home with near-baseline mental capacity for further physical therapy.

## Introduction

Meningitis, an infection of the meninges of the central nervous system (CNS), can advance quickly and carries a mortality rate reaching 30% among affected patients [1]. Meningitis often presents with a vague constellation of symptoms, such as fever, altered sensorium, and neck stiffness, and can affect different regions of the CNS. It may become complicated by conditions, such as hydrocephalus, ventriculitis, and cerebral abscess. Each of these complications has specific radiological signs on imaging and requires more extensive treatment than uncomplicated meningitis. Given the possible debilitating outcomes, meningitis and its complications require a high degree of suspicion for diagnosis and prompt initiation of treatment to improve the outcomes and decrease the high risk of mortality [1-5]. Meningitis risk factors include extremes of age, alcohol use disorder, malignancy, splenectomy, and diabetes mellitus [6,7]. Here, we describe a case of meningitis complicated by pyogenic ventriculitis and hydrocephalus in a patient with hematological malignancy who underwent chemotherapy and radiotherapy.

## Case presentation

An 85-year-old woman with a past medical history of diffuse large B-cell lymphoma (DLBCL) with metastatic involvement of the pelvis and brain, who completed chemo- and radiotherapy three months ago, was admitted to the hospital due to an acute change in mental status. The family gave a history of high-grade fever, with few episodes of non-bloody vomiting, and inability to recognize family members for the past two days. At baseline, the patient exhibited mental alertness and interactivity, albeit with limited physical mobility. The patient's medical history revealed a diagnosis of DLBCL two years prior. Treatment commenced with the R-CHOP regimen (rituximab, cyclophosphamide, doxorubicin, vincristine, and prednisone), followed by four cycles of loncastuximab tesirine-lpyl. In addition, the patient completed a 14-day course of 10 fractions of 30 Gy whole-brain radiotherapy (WBRT) along with radiation therapy for pelvic metastasis.

On admission, she was obtunded, and the history was obtained from the family. Her vital signs were notable for a fever of 102.7, a heart rate in the 120s-130s, and a blood pressure of 130/72 mmHg. Physical examination revealed an ill-appearing elderly female, with regular tachycardic rhythm, and no additional heart sounds. The lung exam demonstrated normal breath sounds bilaterally without wheezing, crackles, and rales. The abdominal examination was unremarkable. Neurologic examination showed equal and reactive pupils, neck and generalized muscle stiffness, and positive Brudzinski sign. Her laboratory data was notable for normal electrolytes, a WBC count of 17.5 with 91% neutrophils, procalcitonin 2.28, and elevated troponins. Urinalysis was positive for leukocyte esterase and 3-5 bacteria. EKG showed normal sinus rhythm. The respiratory panel was positive for rhinovirus. Blood cultures were taken, and a computed tomography (CT) scan of the head followed by a lumbar puncture was performed as there was a high suspicion of meningitis. The CT scan of the head showed disproportionate ventriculomegaly, pansinusitis, and a large left mastoid effusion. A CT scan chest was also performed to evaluate for possible pneumonia but was negative. She was started on acyclovir, vancomycin, cefepime, and 6 mg of dexamethasone as initial treatment.

**Figure 1:**
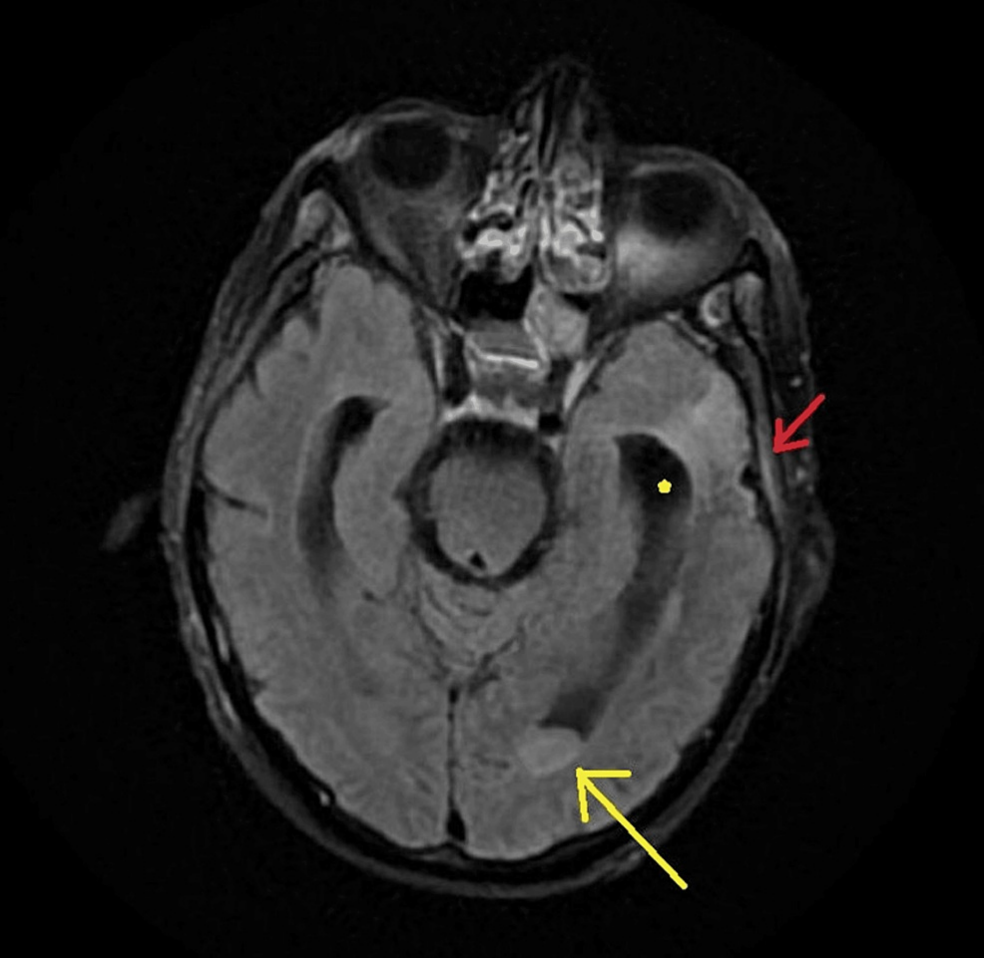
Magnetic resonance imaging fluid-attenuated inversion recovery (MRI FLAIR) showing debris in the posterior horn of the lateral ventricle with enhanced margins (yellow arrow), with ventriculomegaly (yellow star) and meningeal enhancement overlying left parietal lobe (red arrow) indicating meningitis.

The two sets of blood cultures that were obtained during admission subsequently grew *Streptococcus pneumoniae*. The cerebrospinal fluid (CSF) analysis showed hazy slightly xanthochromic fluid with glucose of 49, protein of 287.7, WBCs of 3560 (91% neutrophils), and RBCs of 155. Within hours of lumbar puncture, CSF PCR was positive for *S. pneumoniae* as well. Urine cultures were negative. MRI of the brain showed layering in ventricles, moderate communicating hydrocephalus, and dural enhancement overlying left frontal and parietal lobes consistent with pachymeningitis (Figure 1).

When culture sensitivities became available, the patient's antibiotics were narrowed to ceftriaxone 2 g twice daily. The patient's condition started to improve over the next two to three days. She began to recognize her family and slowly restarted interaction. Her muscular rigidity also lessened. Dexamethasone was discontinued on the fourth day of hospitalization. The patient completed her 21-day course of ceftriaxone, and repeated blood cultures were negative. She was discharged to home on tube feedings after a PEG tube placement, and she was advised to continue home-physical therapy.

## Discussion

Bacterial meningitis is a highly aggressive and devastating CNS infection, with mortality rates ranging from 3% to 30% [1]. The unique physiological characteristics of the brain, such as the absence of lymphatic drainage and the lack of capillaries in the subarachnoid space, coupled with an efficient CSF circulation system, create an ideal environment for bacteria to migrate from infected meninges into the CSF. This migration results in the dissemination of the infection into the subarachnoid space and ventricles [2]. Ventriculitis, an infrequent complication of meningitis, manifests as a purulent infection of the ependymal lining and is also referred to as intraventricular empyema, pyocephalus, or ependymitis. In addition to meningitis, common causes of ventriculitis include intraventricular drains, ruptured brain abscesses, and neurosurgical interventions, particularly in immunocompromised patients with conditions, such as malignancy, alcoholism, and diabetes. The predominant bacterial species associated with ventriculitis include *Streptococcus* (44.9%), gram-negative *Bacillus* (27.6%), and *Staphylococcus* (15.3%) [3,4]. Another rare complication of meningitis is hydrocephalus, with an incidence rate of less than 5%. High mortality in cases of bacterial meningitis-related hydrocephalus is primarily attributable to elevated intracranial pressure, leading to cerebral herniation. Communicating hydrocephalus is more common than obstructive hydrocephalus, with the former resulting from impaired CSF absorption through arachnoid villi and the latter developing gradually due to inadequate treatment, leading to the obstruction of the foramina of Luschka and Magendie [5]. Overall, immunocompromised conditions represent the primary risk factor for bacterial invasion of the CNS [6]. Cancer patients, particularly those with hematological malignancies, often exhibit hypogammaglobulinemia, which heightens susceptibility to infections caused by encapsulated organisms, such as *Streptococcus pneumoniae*, mirroring the situation in our patient. In addition, the administration of chemotherapy and corticosteroids, as observed in our case, exacerbates immunosuppression, further predisposing individuals to invasive CNS infections [6].

The most frequently observed presenting symptoms in patients with meningitis and ventriculitis are high-grade fever (92%), altered mental status (82%), headache (74%), neck stiffness (58%), and photophobia/phonophobia (40%). The classic triad of fever, altered sensorium, and neck rigidity is only present in approximately 33% of cases, a statistic that aligns with our patient's presentation [4]. However, studies have indicated that altered mental status, Kernig's sign, and Brudzinski's sign have limited diagnostic accuracy. Brouwer et al. reported sensitivities of 31%, 11%, and 9%, respectively, and specificities of 71%, 95%, and 95%, respectively [8]. Therefore, a high degree of clinical suspicion must be supplemented with CSF analysis and imaging for a definitive diagnosis.

CSF analysis and culture remain the gold standard for diagnosing bacterial meningitis, with cultures yielding positive results in more than 80% of cases when obtained before treatment initiation [8]. Although performing a head CT before a lumbar puncture is often recommended to exclude signs and causes of elevated intracranial pressure (ICP), its necessity is subject to debate. A retrospective study indicated that a CT scan before lumbar puncture resulted in a delay of more than six hours in initiating antimicrobial treatment for 63% of patients. This delay has the potential to critically change the outcome [9]. However, certain risk factors and clinical signs have been identified, and in their absence, a pre-lumbar puncture CT scan may not be required. Risk factors for elevated ICP include advanced age, CNS disease, space-occupying brain lesion, and an immunocompromised state, while indicative clinical signs include focal neurological deficits, new-onset seizures, papilledema, and moderate to severe impairment of consciousness. Due to a history of brain metastatic lesions, a head CT scan was warranted in our patient. Neuroradiological signs suggestive of elevated ICP include coning (herniation of cerebellar tonsils through the foramen magnum), effacement of basal cisterns, ventricles, and sulci, and the cerebellar reversal sign (a relatively enhanced appearance of the normal cerebellum compared to hypodense anoxic supratentorial brain) [8,10].

Characteristic findings in CSF analysis for meningitis and ventriculitis include polymorphonuclear pleocytosis (greater than 50%), hypoglycorrhachia (less than 25 mg/dL), and elevated CSF protein levels (greater than 50 mg/dL). Although CSF culture is the standard for identifying causative organisms, nucleic acid amplification tests such as PCR have demonstrated higher sensitivity and specificity, exceeding 80% and 95%, respectively. These tests can also provide results within as little as two hours [4,8]. Kong et al. reported that heparin-binding protein (HBP), released from activated neutrophils, displayed greater diagnostic accuracy compared to lactate and procalcitonin, with levels remaining elevated (greater than 23 ng/mL) even in patients treated with antimicrobials for more than 48 hours. This is identified as a reliable adjunctive diagnostic marker [11].

Neuroimaging plays a crucial role in confirming the diagnosis of meningitis, detecting complications, monitoring treatment progress, and, as previously mentioned, ruling out elevated ICP before lumbar puncture. In cases of uncomplicated meningitis, contrast-enhanced CT scans of the brain can appear normal. MRI FLAIR and T2-weighted images may reveal hyperintense subarachnoid spaces, particularly along the cerebral convexity. Atypical and chronic forms of meningitis may exhibit more prominent abnormalities in the basal cisterns [2]. Occasionally, the subcortical white matter may appear hypointense due to disrupted oxygen-free radical systems beneath inflamed meninges. Common indicators of ventriculitis on MRI FLAIR include ependymal enhancement, hyperintense fluid levels (pus) within the ventricles, and occasionally intraventricular septations. Elderly patients are more susceptible to developing hydrocephalus as a complication, with third ventricle or temporal horn dilatation being the most sensitive indicator [4,10,12].

In patients over the age of 50, empiric antimicrobial therapy involving vancomycin, ampicillin, third-generation cephalosporin, and acyclovir is recommended [13]. In our patient, *S. pneumonia *was cultured from blood, prompting the initiation of treatment with intravenous ceftriaxone. Although no set guidelines dictate the duration of antibiotic therapy for meningitis and ventriculitis, the general consensus is 10-14 days for uncomplicated meningitis and six to 12 weeks for ventriculitis [3,13]. The continued administration of dexamethasone is also advised to prevent neurological sequelae and hearing loss, particularly in cases of *Haemophilus influenzae* or *S. pneumoniae* meningitis [13]. In cases where signs of increased intracranial pressure are evident, patients with hydrocephalus or ventriculitis complications may require an external ventricular drain or a shunt placement. In our patient's case, these interventions were unnecessary, as there was a low ICP during lumbar puncture. Clinical improvement was observed after three weeks of antibiotic treatment [4].

## Conclusions

In immunocompromised patients, especially in the setting of malignancy, acute change in mental status with fever should raise high suspicion of meningitis. In such situations, a physical examination aids in the diagnosis, which CSF fluid investigations can confirm. Clinicians should bear in mind the infection and sequela involving other parts of the central nervous system, which demands prolonged antimicrobial course and potentially invasive procedures. Pyogenic ventriculitis is an uncommon yet severe complication of bacterial meningitis that can result in ventricle obstruction if left untreated. Hydrocephalus symptoms are generally visible, but they can also be clinically quiet. Although vast academic data on the presentation of CNS infection is available, in clinical settings, events of misdiagnosis or delayed diagnosis can occur due to diverse clinical presentations, which often can lead to deleterious consequences. Initial treatment with broad-spectrum antibiotics with adequate CNS penetration and repeat imaging to rule out complications should be performed if symptoms increase or continue after adequate treatment.

## References

[1] Ribeiro B, Bishop P, Jalili S (2020). When a stroke is not just a stroke: Escherichia coli meningitis with ventriculitis and vasculitis: a case report. J Crit Care Med (Targu Mures).

[2] Guerra M, Marado D, Fortuna J (2023). Acute meningitis complicated with ventriculitis caused by Streptococcus dysgalactiae subsp. dysgalactiae. Arch Clin Cases.

[3] Maheshwarappa HM, Rai AV (2022). A rare case of primary pyogenic ventriculitis in a patient with community-acquired meningitis. Indian J Crit Care Med.

[4] Sakurai M, Watari T, Nakamura I, Azuma K, Homma H (2022). Acute hydrocephalus associated with Streptococcus anginosus meningitis. Eur J Case Rep Intern Med.

[5] Coelho E, Costa L, Martins J, Costa M, Oliveira JE, Maia-Gonçalves A, Lencastre L (2021). Healthcare-associated ventriculitis and meningitis: a retrospective analysis. Cureus.

[6] Baden LR, Swaminathan S, Angarone M (2016). Prevention and treatment of cancer-related infections, version 2.2016, NCCN clinical practice guidelines in oncology. J Natl Compr Canc Netw.

[7] Costerus JM, Brouwer MC, van der Ende A, van de Beek D (2016). Community-acquired bacterial meningitis in adults with cancer or a history of cancer. Neurology.

[8] Cahill JA, Li C, Wong PH (2022). Group B streptococcal leptomeningitis, ventriculitis, right cerebellitis, and cerebritis in an immunocompetent patient. J Assoc Med Microbiol Infect Dis Can.

[9] Luque-Paz D, Revest M, Eugène F, Boukthir S, Dejoies L, Tattevin P, Le Reste PJ (2021). Ventriculitis: a severe complication of central nervous system infections. Open Forum Infect Dis.

[10] Meguins LC, Rocha AS, Laurenti MR, de Morais DF (2021). Ventricular empyema associated with severe pyogenic meningitis in COVID-19 adult patient: case report. Surg Neurol Int.

[11] Adhikari P, Antala D, Pyakuryal B, Muhammed A, Pudasainee P, Friedman H, Ezepue CJ (2022). Community-acquired meningitis complicated by pyogenic ventriculitis: a case report. Cureus.

[12] Kong Y, Ye Y, Ma J, Shi G (2022). Accuracy of heparin-binding protein for the diagnosis of nosocomial meningitis and ventriculitis. Crit Care.

[13] Duong MT, Rudie JD, Mohan S (2023). Neuroimaging patterns of intracranial infections: meningitis, cerebritis, and their complications. Neuroimaging Clin N Am.

